# Frequency of Diagnostic Classification Systems’ Usage by Mental Health Professionals in Day-to-Day Clinical Practice

**DOI:** 10.1192/bjo.2022.257

**Published:** 2022-06-20

**Authors:** Eleni Vrigkou, Robert Stamatakis, Katja Umla-Runge

**Affiliations:** 1 Centre for Medical Education, Cardiff University, United Kingdom; 2 The Caswell Clinic, Swansea Bay, United Kingdom

## Abstract

**Aims:**

Diagnostic classification systems (DCSs) are medical models constructed by experts with the purpose of facilitating diagnostic processes. Specifically in psychiatry, DCSs serve as mental health professionals’ major diagnostic tool. Several studies, however, suggest that mental health professionals may not systematically apply the DCSs in day-to-day practice. The primary aim of this secondary research was to assess the actual frequency of DCSs’ application in psychiatric practice. All DCSs were considered. The secondary aims were to investigate the mode of DCSs’ application (e.g., assign diagnosis, inform treatment, administrative/billing or teaching purposes), and to assess if DCSs’ usage patterns vary depending on the clinicians’ specific occupation (e.g., psychiatrists, psychologists) and country of employment.

**Methods:**

The bibliographic databases of MEDLINE Via Ovid, PsycInfo, Web of Science and Global Health were searched from 1/2000–12/2020. All primary studies assessing DCSs’ frequency of application by mental health professionals were eligible for inclusion. The search yielded nine eligible articles. The total number of participants from all included studies was 10,388. The study samples were diverse, including practitioners from a wide variety of geographical locations, languages, and income-level countries.

**Results:**

The results of the study showed that 69% (95%CI = 58–80%) of the responders use DCSs “often, almost always or always” in day-to-day practice. Regarding the mode of DCSs’ application, responders stated that they use DCSs most frequently for administrative/billing purposes and assigning a diagnosis. The study's results also showed that 68% (95%CI = 45–90%) of psychiatrists and 74% (95%CI = 43–100%) of psychologists use the DCSs “often, almost always or always”. Subgroup analysis based on responders’ country of employment suggest that the frequency of “often, almost always or always” DCSs’ usage (according to World Health Organization regions) were: for the Region of the Americas 75.3%, for the African Region 73.5%, for the Western Pacific Region 71.6%, for the European Region 69.4%, for the South-East Asia Region 66.8%, and for the Eastern Mediterranean Region 57.1%.

**Conclusion:**

The study's outcomes indicate that DCSs are integrated into the daily practices of mental health professionals worldwide. Further research is needed, however, in order to assess in more depth DCSs’ application practices (e.g., comparative usage of different DCSs, types of mental disorders, patients and settings where DCSs are more frequently applied). Such findings could be valuable, since they can be used to help appraise the quality of DCSs’ actual use, the impact of DCSs on clinical care and public health, and also to aid design more effective mechanisms for DCSs’ further implementation.

